# Annular Pancreatitis and Gastric Cancer

**DOI:** 10.1155/1993/57374

**Published:** 1993

**Authors:** S. B. Kelly, R. C. N. Williamson

**Affiliations:** Department of Surgery Royal Postgraduate Medical School Hammersmith Hospital Du Cane Road London W12 ONN UK

## Abstract

Annular pancreas can present in adult life, usually when inflammation of the pancreatic ring causes duodenal stenosis. Although peptic ulcer is often associated with annular pancreas, an association with gastric carcinoma has not been previously described. Here we report a patient with annular pancreas, chronic alcoholic pancreatitis and gastric carcinoma, who was successfully treated by radical proximal pancreatoduodenectomy.


*HPB Surgery*, 1993, Vol. 7, pp. 69–75
Reprints available directly from the publisher
Photocopying permitted by license only

© 1993 Harwood Academic Publishers GmbH
Printed in the United States of America

### CASE REPORT

### ANNULAR PANCREATITIS AND GASTRIC CANCER

S. B. KELLY and R.C.N. WILLIAMSON*
*Department of Surgery, Royal Postgraduate Medical School, Hammersmith Hospital, Du Cane Road, London W12 ONN, UK*

*(Received 13 December 1991)*

Annular pancreas can present in adult life, usually when inflammation of the pancreatic ring causes duodenal stenosis. Although peptic ulcer is often associated with annular pancreas, an association with gastric carcinoma has not been previously described. Here we report a patient with annular pancreas, chronic alcoholic pancreatitis and gastric carcinoma, who was successfully treated by radical proximal pancreatoduodenectomy.

KEY WORDS: Pancreas, pancreatitis, stomach neoplasms

### INTRODUCTION

The congenital anomaly of a ring of pancreas around the duodenum was described by Tiedemann in 1818[1] and termed annular pancreas by Ecker in 1862[2]. About half the individuals destined to develop symptoms from annular pancreas do so within a year of birth, one third of them during the first week of life. In 85% of cases the pancreatic annulus encircles the second part of the duodenum, but the first part or, rarely, the third part may also be involved[3]. The obstruction is nearly always proximal to the ampulla, so that the infant vomits clear juice devoid of bile.

Presentation in adult life usually results from inflammatory change in the ectopic pancreatic tissue[4]. There is progressive narrowing of the duodenal lumen and concomitant hypertrophy of the proximal duodenal wall[5]. Peptic ulcer is a frequent complication, but gastric carcinoma does not appear to have been described previously in a patient with this congenital anomaly. We report a middle-aged man with chronic alcoholic pancreatitis (involving the pancreatic head and annulus) plus gastric cancer, in whom an extended Whipple resection has been curative.

### CASE REPORT

A 43-year-old man presented with a nine month history of epigastric pain radiating

*Address correspondence to: Professor R.C.N. Williamson, Department of Surgery, Royal Postgraduate Medical School, Hammersmith Hospital, Du Cane Road, London, W12 0NN, UK

to the back, intermittent vomiting and 12 kg weight loss. He had mild indigestion for five years. He worked for a brewery and was a heavy beer drinker (7.5l/day for 20 years). Physical examination was normal, together with serum amylase, glucose tolerance test and intravenous cholangiogram; stools were negative for occult blood. There were modest elevations in serum bilirubin (23 $\mu$mol/l; normal $< 17$), aspartate aminotransferase (78 iu/l; normal $< 17$) and three-day faecal fat excretion (22.3 mmol/day; normal $< 15$). Barium meal and endoscopy both showed an extrinsic lesion surrounding the second part of the duodenum (Figure 1); the papilla could not be seen. The stomach appeared normal apart from a 1 cm antral polyp. CT scan demonstrated a moderately large mass in the region of the head of the pancreas (Figure 2). Angiography showed "cut-off" of the inferior pancreaticoduodenal artery but no tumour blush. Preoperative diagnoses included pancreatic cancer, duodenal cancer, chronic pancreatitis and annular pancreas.

At laparotomy, the head of pancreas was swollen, indurated and inflamed with less severe changes of pancreatitis in the distal gland. In addition, there was a nodular mass high on the lesser curve of the stomach (which had been missed on the preoperative tests) (Figure 3). Proximal pancreatoduodenectomy was performed together with 80% distal gastrectomy and cholecystectomy. Reconstruction was by an end-to-end pancreatojejunostomy with choledochojejunostomy and gastrojejunostomy downstream.

Macroscopic examination of the resected specimen revealed a circumferential constricting lesion involving the second part of the duodenum (Figure 4). This thickened rim of tissue was opened along its length to reveal a large duct (8mm diameter), which encircled the duodenum and communicated with the remainder of the pancreatic ductal tree. The common bile duct appeared entirely normal in its intrapancreatic portion.

Histological examination showed severe chronic pancreatitis in the head of pancreas and annulus with acute inflammatory change and abscess formation. The distal pancreas was normal. The ulcerating lesion in the stomach was a moderately differentiated adenocarcinoma of intestinal type, which had invaded but not penetrated the muscle coat. An enlarged lymph node showed reactive change only, and a liver biopsy showed moderate steatosis.

Postoperatively he developed a wound abscess but otherwise recovered satisfactorily. Although he was slow to gain weight, glucose tolerance test and 3-day fecal fat excretion were normal. Four years after operation he was reinvestigated for malaise, weight loss, dyspnoea and chest infection. Raised liver enzymes, low serum albumin and peripheral macrocytosis suggested alcoholic hepatitis, a diagnosis that was confirmed by liver biopsy. There was now evidence of pancreatic exocrine insufficiency, consistent with continuing pancreatitis and/or failure of the pancreatic anastomosis. He improved after reduction of alcohol intake, pancreatic enzyme therapy and introduction of a diet high in protein and carbohydrate but low in fat. Subsequent problems included bile gastritis and a left iliac fossa abscess, considered diverticular in origin. Eleven years after the operation he remains well. His weight is steady and his bowels are regular on Nutrizym (9/day). There is no evidence of recurrent gastric cancer.

### *Discussion*

This is a case of annular pancreas which remained silent until the fifth decade of

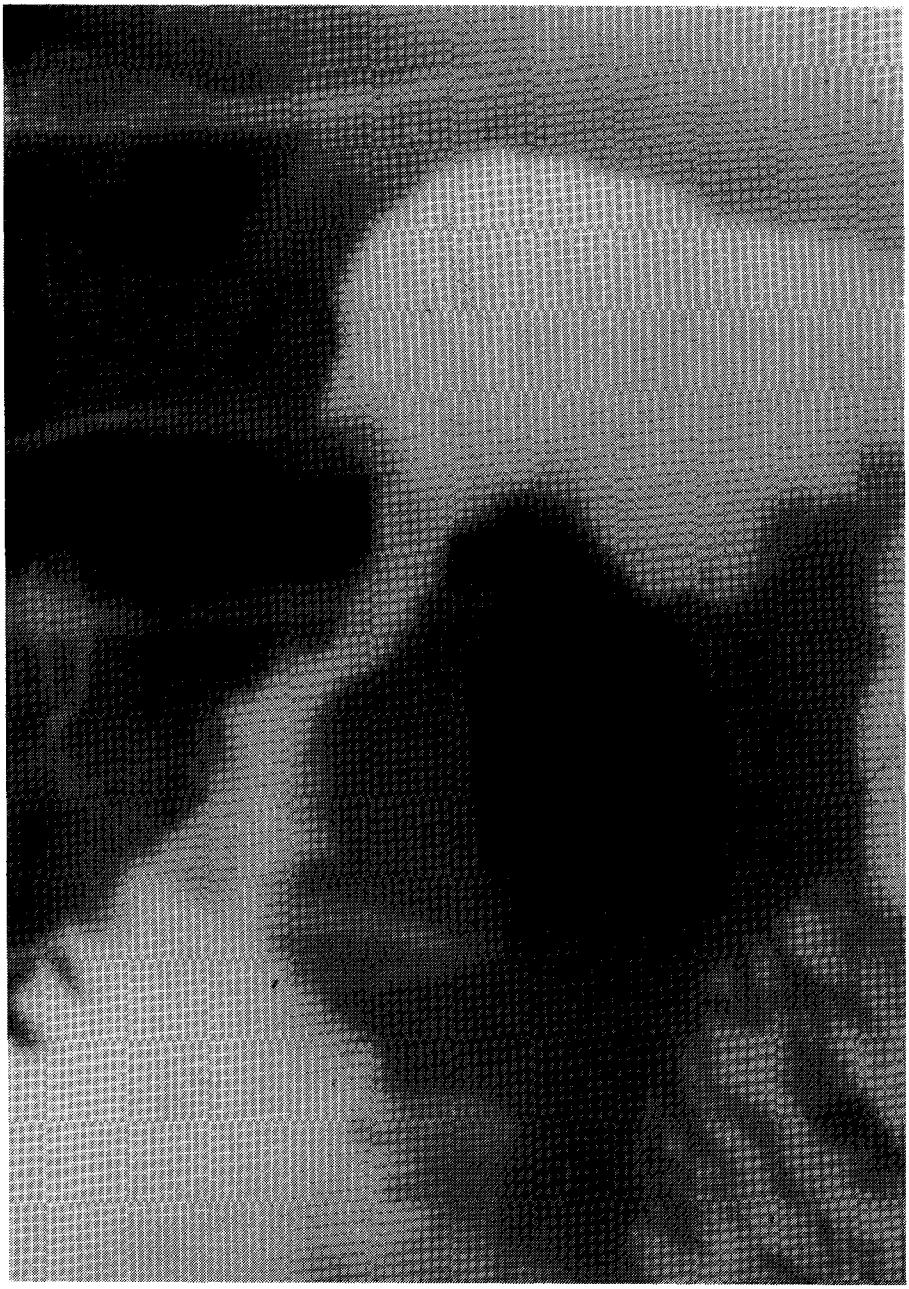

**Figure 1** Barium meal showing gross narrowing of the second part of the duodenum.

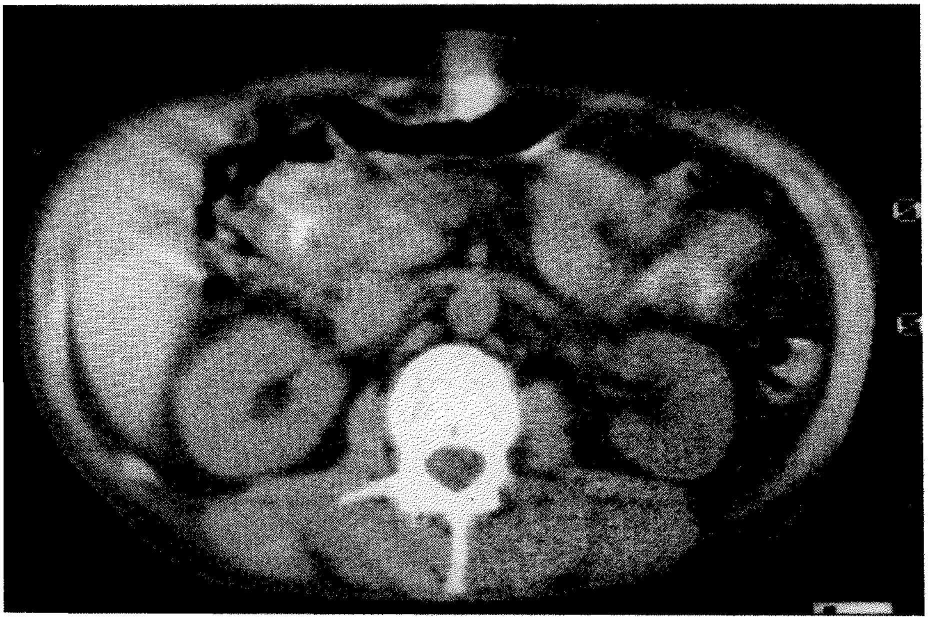

**Figure 2** CT scan demonstrating a moderately large mass containing contrast medium in the region of the head of pancreas.

life, when acute-on-chronic alcoholic pancreatitis led to duodenal obstruction. Inflammatory change within the annulus is a well established complication[4] but here the pancreatitis extended widely into the head of the gland, which contained an abscess. Presumably the extent and severity of disease are explained by the extra aetiological agent, alcohol. The aetiology of pancreatitis in association with annular pancreas is not known, but it may be related to poor drainage of the pancreas due to duodenal stenosis caused by the pancreatic ring[4].

In almost all symptomatic patients with annular pancreas, the ectopic pancreatic tissue completely encircles the gut and lies within the muscle layers of the duodenal wall, as it did in the present case[6]. Embryological development of the pancreas is from two entodermal outpouchings, which fuse during the fourth week of intrauterine life. The dorsal anlage forms the body and tail of the pancreas. The ventral anlage consists of a left portion, which soon atrophies, and a right portion, which contributes to the head and neck of the pancreas. Duodenal rotation brings the ventral anlage to lie adjacent to its dorsal counterpart by the eighth week of gestation. The formation of an annulus of pancreatic tissue probably occurs by the following mechanism: as the left ventral bud atrophies, the tip of the right ventral bud becomes adherent to the anterior part of the duodenum, and a stretched portion of pancreas is left after rotation and elongation of its remaining portion[7]. Associated anomalies can include Down's syndrome, nonrotation or incomplete rotation of the mesentery[4], imperforate anus, oesophageal atresia with tracheo-oesophageal fistula and congenital heart disease.

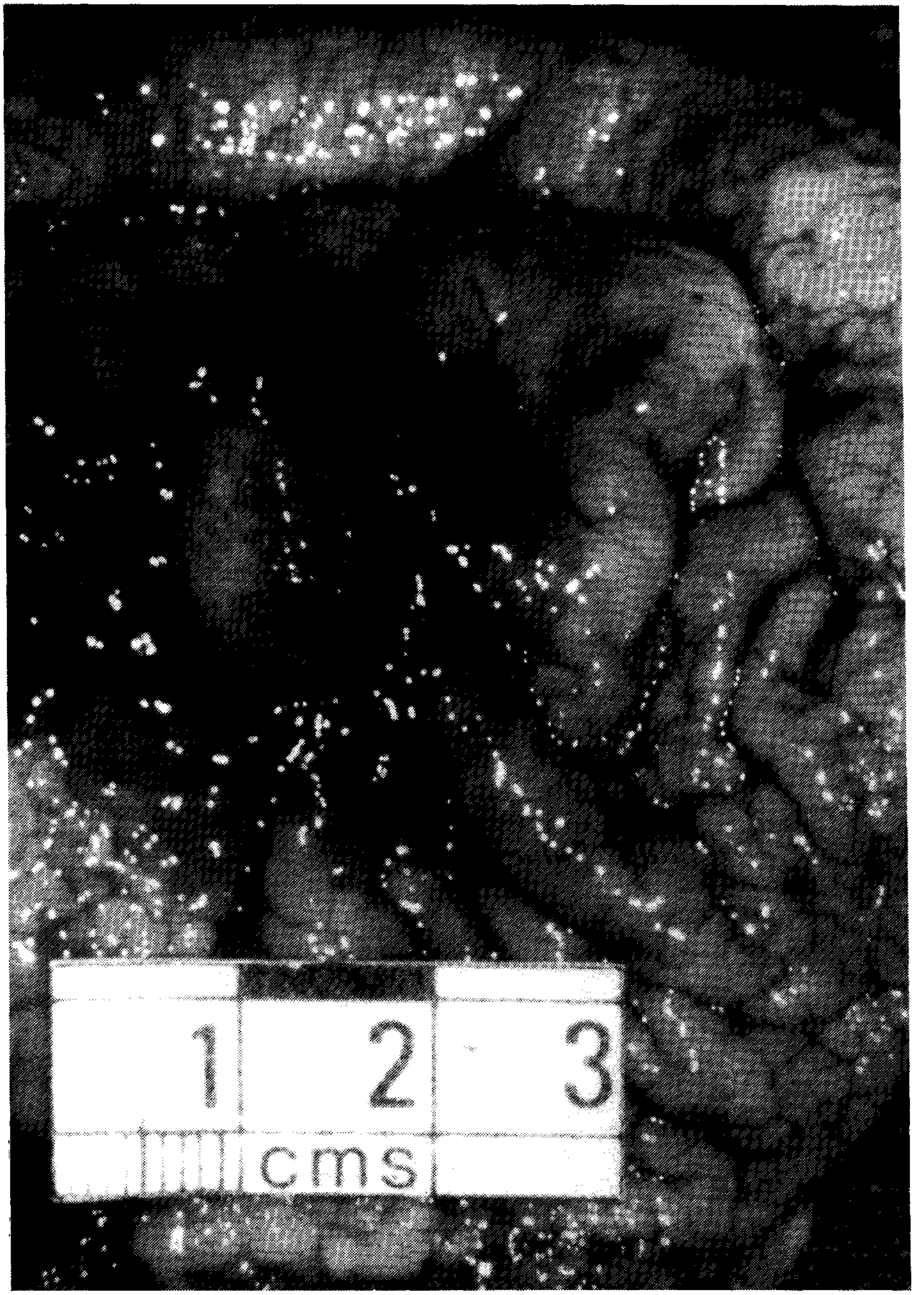

**Figure 3** Portion of opened stomach showing an ulcer measuring 2 × 1 cm, located 3 cm from the proximal resection margin, with slightly raised edges and a haemorrhagic periphery.

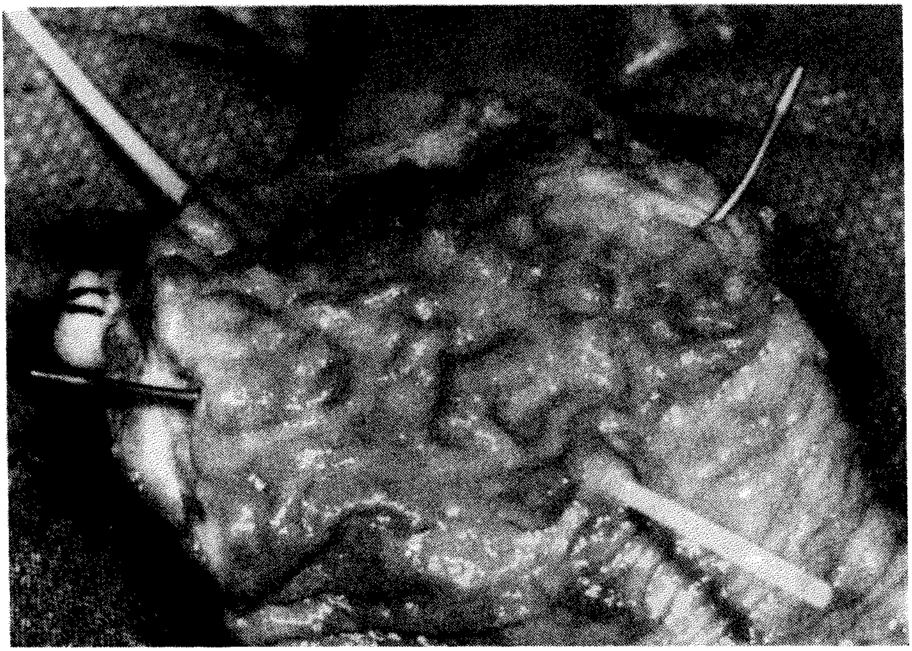

**Figure 4** Resected specimen of duodenum showing a probe in the annular duct and a catheter in the common bile duct.

Peptic ulcer disease has been reported in some 25% of adults with annular pancreas[8,9]. It could reflect hypersecretion of acid by the obstructed stomach or reduced access of alkaline secretions (bile and pancreatic juice) to the proximal duodenum. Gastric carcinoma has not previously been reported in annular pancreas to our knowledge. Conceivably it might have arisen by malignant transformation of a gastric ulcer; in support, there was a 5-year history of dyspepsia and peptic ulcer disease is common among alcoholics. Alternatively, chronic gastric stasis allowed bacterial overgrowth which led in turn to endogenous nitrosation and formation of intraluminal carcinogens. Discovery of the carcinoma at a relatively early stage was fortuitous. The tumour, which lay high up on the anterior wall of the stomach, had been missed on repeated preoperative barium studies and endoscopy. Five-year survival rates of up to 50% may be anticipated for cancers confined to the stomach wall[10], and eleven-year survival may represent cure.

Vomiting is the principal symptom associated with annular pancreas, irrespective of age. Jaundice is an infrequent complication, probably because the annulus lies above the level of the papilla[11]. In one series of neonates there was an 18% incidence of jaundice[3]. Our patient had minimal elevation of serum bilirubin.

In duodenal obstruction apparently caused by annular pancreas, surgical treatment must take account of the probable presence of an annular pancreatic duct[12] or even the common bile duct[13] within the encircling pancreatic tissue. Simple division of the pancreatic ring would often be difficult because of its intramural course (as in our patient). In any case it is fraught with complications: acute pancreatitis, pancreatic fistula and incomplete relief of the obstruction. A bypass operation is therefore preferred, either a duodenoduodenostomy or duodenojejunostomy in infancy or gastrojejunostomy (usually with vagotomy), especially in elderly patients who have a lesser risk of stomal ulceration.

The severity of inflammatory disease in the present case (and the possibility of pancreatic cancer) led us to perform proximal pancreatoduodenectomy. The additional presence of a high gastric cancer necessitated subtotal gastrectomy. Despite continuing alcoholism, early cirrhosis and pancreatic exocrine failure, the patient remains in satisfactory health and nutritional status eleven years later.

*References*

1. Tiedemann, F. (1818) Uber die Verschiedenheiten des Ausfuhrungsganges der Bauchspeicheldruse dei den Menschen und Saugetieren. *Dtsch. Arch. Physiol.*, **4**, 403
2. Ecker, A. (1862) Bildungsfehier des Pankreas und des Herzens. *Z. Art. Med.*, **14**, 354
3. Peiper, H.J. and Muller-Heubach, E. (1968) Das Pankreas anulare und seine chirurgische Behandlung. *Langenbecks Arch. Clin. Chir.*, **320**, 322–354
4. Ravitch, M.M. and Woods, A.C. Jr. (1950) Annular pancreas. *Ann. Surg.*, **132**, 6
5. Ravitch, M.M. (1975) The pancreas in infants and children. *Surg. Clin. N. Amer.*, **55**, 377–385
6. Hyden, W.H. (1963) The true nature of annular pancreas. *Ann. Surg.*, **157**, 71–77
7. Lecco, T.M. (1910) Zur Morphologie des Pankreas annulare. *Sitzungsb. Akad. Wissensch.*, **119**, 391–406
8. Kiernan, P.D., ReMine, S.G., Kierman, P.C., ReMine, W.H. (1980) Annular Pancreas. *Arch. Surg.*, **115**, 46–50
9. Drey, N.W. (1957) Symptomatic annular pancreas in the adult. *Ann. Int. Med.*, **6**, 750–772
10. Humphrey, C.S. and Cuschieri, A. (1988) The stomach and duodenum. In: *Essential Surgical Practice*, edited by A. Cuschieri, G.R. Giles and A.R. Moossa, 2nd edition pp. 955–988. London: Wright
11. Dodd, G.D. and Nafis, W.A. (1956) Annular pancreas in the adult. *Am. J. Roentgenol.*, **75**, 333–342
12. Heymann, R.L. and Whelan, T.J. Jr. (1967) Annular pancreas. Demonstration of the annular duct on cholangiography. *Ann. Surg.*, **165**, 470–472
13. Anderson, J.R. and Wapshaw, H. (1951) Annular pancreas. *Br. J. Surg.*, **39**, 43–49

### INVITED COMMENTARY

This is an exceptional case report of annular pancreas and the combination with a gastric cancer. The gastric cancer was not diagnosed before the operation. Because of an uncertain mass in the pancreatic head, signs of severe pancreatitis and the suspicion of a possible pancreatic carcinoma the decision of a proximal pancreatoduodenectomy was justified and the best solution. There are interesting points of discussion concerning the possible etiology of the gastric cancer with regard to the duodenal stenosis by an annular pancreas.

The authors theory of a possible cancer development on the ground of a chronic gastric ulcer in the sense of a Dragstedt combination seems logical and can be accepted.

H. J. Peiper

3400 Göttingen

Germany

